# A Method for the Analysis of African Swine Fever by Viral Metagenomic Sequencing

**DOI:** 10.3389/fvets.2021.766533

**Published:** 2021-11-23

**Authors:** ChiHai Ji, JingZhe Jiang, YingFang Wei, ZhiYuan Wang, YongJie Chen, ZhanZhuo Mai, MengKai Cai, ChenXiao Qin, Yu Cai, HeYou Yi, Guan Liang, Gang Lu, Lang Gong, GuiHong Zhang, Heng Wang

**Affiliations:** ^1^Guangdong Provincial Key Laboratory of Prevention and Control for Severe Clinical Animal Diseases, College of Veterinary Medicine, South China Agricultural University, Guangzhou, China; ^2^Shenzhen Kingkey Smart Agriculture Times Co., Ltd., Shenzhen, China; ^3^Key Laboratory of Zoonosis Prevention and Control of Guangdong Province, Guangzhou, China; ^4^Guangdong Laboratory for Lingnan Modern Agriculture, Guangzhou, China; ^5^National Engineering Research Center for Breeding Swine Industry, Guangzhou, China; ^6^Key Laboratory of South China Sea Fishery Resources Exploitation and Utilization, Ministry of Agriculture, South China Sea Fisheries Research Institute, Chinese Academy of Fishery Sciences, Guangzhou, China; ^7^Guangdong Meizhou Vocational and Technical College, Meizhou, China

**Keywords:** African swine fever virus (ASFV), genome, virome, virus-like particle, whole genome sequencing

## Abstract

In 2018, there was an outbreak of African swine fever (ASF) in China, which spread to other provinces in the following 3 years and severely damaged China's pig industry. ASF is caused by the African swine fever virus (ASFV). Given that the genome of the African swine fever virus is very complex and whole genome information is currently inadequate, it is important to efficiently obtain virus genome sequences for genomic and epidemiological studies. The prevalent ASFV strains have low genetic variability; therefore, whole genome sequencing analysis provides a basis for the study of ASFV. We provide a method for the efficient sequencing of whole genomes, which requires only a small number of tissues. The database construction method was selected according to the genomic types of ASFV, and the whole ASFV genome was obtained through data filtering, host sequence removal, virus classification, data assembly, virus sequence identification, statistical analysis, gene prediction, and functional analysis. Our proposed method will facilitate ASFV genome sequencing and novel virus discovery.

## Introduction

ASF is a highly contagious and fatal swine disease. The pathogen of ASF is the African swine fever virus (ASFV), which is the only member of the family *Asfarviridae* and the only known DNA arbovirus (1, 2). Depending on the strain, ASFV has a large (170–193 kbp) double-stranded DNA genome containing 151–167 genes, which are involved in viral replication and assembly as well as in modulating host cellular functions and immune evasion (3). The virus can be transmitted through direct contact with infected swine, their products, and soft ticks of the genus *Ornithodoros* (4). ASF was first described in Kenya in 1921; it then spread to other African, European, Caribbean, and South American countries (3, 5, 6). The disease was introduced into Georgia in 2007 and then spread throughout Eastern Europe, including Russia, Belarus, Ukraine, Estonia, Lithuania, Latvia, Romania, Moldova, Czech Republic, and Poland (7–9). In August 2018, China reported its first ASF outbreak. Within 1 year, ASFV spread rapidly into all provinces in mainland China. The spread of ASF has resulted in huge losses to the Chinese pig industry (3).

ASFV has a high genetic and antigenic diversity. Based on the p72 protein (B646L), 24 genotypes have been identified, while at least eight serotypes are recognized based on hemadsorption inhibition (10, 11). The spread of the African swine fever virus in China is a serious threat to the diversity and survival of pigs. To facilitate much needed epidemiological investigations, advance research, and further vaccine development, it would be expedient to have a simple and reproducible method for full genome sequencing of the ASFV (12). In the early stage, we used the first-generation sequencing technology to sequence the whole genome of ASFV, which is very time-consuming and with heavy workload. This demands for a faster method for rapid sequencing of the whole ASFV genome, and second-generation sequencing is an important tool for sequencing large genomes, which is essential for effective emergency management in the event of disease outbreaks. Current methods of virus enrichment in second-generation sequencing are inefficient and time-consuming. By improving the enrichment method, we can effectively increase the proportion of virus samples and provide more effective data for subsequent analysis. As the ASFV genome contains a wide range of homopolymers and repeat regions, the short-read data generated by second generation sequencing platforms need to be processed carefully. ASFV genome recombination (such as inversion or duplication) may be missed when comparing reference sequences, and the quality of the consistent sequence is heavily dependent on the reference sequence and therefore, may be misassembled. After sequencing, data filtration, host sequence removal, virus classification, data assembly, virus sequence identification, statistical analysis of virus abundance, gene prediction, and functional analysis were performed. Through the analysis of these steps, an accurate sequence is finally obtained. Our results confirm the feasibility of sequencing an ASFV genome directly from positive clinical tissues, and provide a basis for further epidemiological research and evolutionary analysis (13).

## Materials and Methods

### Experimental Materials

We obtained 0.45 μm and 0.22 μm filter (PVDF) membranes from Merck Display Materials Co., Ltd. (Shanghai, China), OptiPrep Density Gradient Medium purchasedfrom Guangzhou Fan-si Biotechnology Co., Ltd. (Guangzhou, ChinaGuangzhou, ChinaGuangzhou, ChinaGuangzhou, ChinaGuangzhou, China), DNase I (Promega Corporation, Madison, USA), BSA (configured as 1% BSA-SM solution, filtered by 0.22 μm membrane), and gelatin from porcine skin purchased from Sigma-Aldrich Trading Co., Ltd. (Shanghai, China). Furthermore, we used an overspeed centrifuge (Beckman Coulter, USA), HiPure Viral DNA Kit (D3191) purchased from Majorbio (Shanghai, China), and 10 × SM buffer (pH 7.5, 1 M NaCl, 100 mM MgSO4, 500 mM Tris, 0.1% gelatin; working concentration was diluted with ultra-pure water to 1 × SM). Op density gradient solution was made using Optiprep original solution, mixed with 10 × SM buffer (9:1).

### Enrichment and Purification of Virus-Like Particles (VLPs)

Cases were identified from the Ministry of Agriculture and Rural Affairs of Huangpu District, Guangzhou (information released by the Ministry of Agriculture and Rural Affairs: http://www.moa.gov.cn/gk/yjgl_1/yqfb/201812/t20181223_6165395.htm). The spleen tissue was completely fixed with 10% formalin solution for 72 h. Two formalin fixed pig spleens 1 g each were taking and cut into small pieces with sterile scissors, and then poured into 2 × sucrose -Triton washing solution for washing, 1,000 r/min centrifugation for 15 min, precipitation was beaten evenly with TE Buffer (pH 9.0), adding SDS to the final concentration of 1%, protease K to 200 μg/mL, and kept in water bath at 48°C for 48 h. And 5–10 mL of pre-cooled 1 × SM buffer (SM buffer filtered by 0.22 μm) was added. The spleen was evenly homogenized and placed in liquid nitrogen and a 37°C water bath alternately three times. Spleens were centrifuged at 4°C for 5 min at 1,000 rpm and 12,000 rpm in succession, and the supernatant was filtered using a 0.45 μm filter. Before using the 0.45 μm filter, 1% BSA solution was filtered through the wetting membrane. Then, qPCR was performed on each liquid layer using specific ASFV primers to determine the virus Cq value in each liquid layer. The liquid layer with the low Cq value was selected, each two liquid layers were mixed, and 1 × μSM buffer solution was added to fully mix. The solution was centrifuged at 160,000 *g* for 1 h, and the supernatant was discarded. According to the amount of precipitation, 100–500 μL 1 × SM buffer solution was added for resuspension (with repeated pipette mixing to avoid violent shock). DNaseI was added according to the manufacturer's instructions, with treatment at 37°C for 1 h. Single- and double-stranded DNA were excised simultaneously to fragment the DNA for library construction. EDTA (Promega Corporation, Madison, USA) was added according to the manufacturer's instructions to terminate the reaction. The HiPure Viral DNA Kit (D3191) (Majorbio, Shanghai, China) was used to extract viral DNA, and the concentration was measured. Sequencing libraries were generated using NEB Next® Ultra™ DNA Library Prep Kit for Illumina® (New England Biolabs, MA, USA) following manufacturer's recommendations and index codes were added. Finally, the library was sequenced on an Illumina Novaseq 6,000 and 150 bp paired-end reads were generated. Each sample added 507,333,357 reads. (The extracted DNA was sent to China Guangdong Magigene Technology Co., Ltd [Guangdong, China]) for sequencing. All the experiments involving the ASF virus were carried out in a biosafety level (BSL)-3 laboratory at South China Agricultural University (Guangzhou, ChinaGuangzhou, ChinaGuangzhou, ChinaGuangzhou, ChinaGuangzhou, China).

### Data Filtering

After obtaining the metagenomic sequencing data of the sample, it was necessary to evaluate the quality of the sequencing data and remove low-quality data to ensure the credibility of the subsequent analysis results. High-quality sequences obtained by quality control were used for downstream data analysis. The quality control process used the software SOAPnuke (14), with the specific processing steps as follows: (1) Removal of Adapter Paired reads; (2) Removal of single-ended reads with N's (N, uncertain base information) and >5% of paired reads; (3) When the single-ended sequencing read was low quality (sQ ≤ 20) and when the number of bases was >20% of the total number of read bases, these paired reads were removed; (4) Replicated reads produced by PCR amplification were removed; (5) Removal of polyX (ATCG) sequences.

### Removal of Host Sequences

SOAPaligner (15) and BWA (16) software were used to compare clean reads to the specified host genome and to remove host sequences.

### Virus Classification

The comparison software SOAPaligner (15) and BWA (16) were used to compare clean reads to the virus reference database in order to quickly obtain virus classification information in the samples.

### Data Assembly

The assembly software IDBA (17), SPAdes (18), metaSPAdes, MEGAHIT (19), and Trinity (20) were used to assemble high-quality reads of each sample to obtain a longer contig sequence. Specific software information is provided in the Results section. Then, the number, length, and N50 statistic of the assembly sequence were counted. BWA software was used to compare high-quality reads to the assembly sequence to calculate the utilization rate of assembled reads, and the assembly effect was evaluated using these statistical data.

### Virus Sequence Identification

A variety of methods [including BLAST, HMMSearch (21), and Metagenemark (22)] and databases [including NT, NR, VPFS (23), VFam (24), PFAM (25), and KEGG] were used to identify viral sequences. Annotation was based on the reference database; the corresponding virus sequences were isolated from the NT database, and BLASTN was used to compare contigs with the constructed virus database for species annotation. Using novel virus identification methods to find candidate virus sequences, contigs were compared with multiple databases, as long as one of the following three conditions were satisfied: (1) Comparison between BLASTN (v2.9.0+) and the virus database isolated from NT (virus-NT, including phages) was used to screen the comparison results with *e* ≤ 1 × 10^−5^ (e: exponent); (2) Comparison between BLASTX (v2.9.0+) and the virus-NR database isolated from NR (including bacteriophages) was used to screen the comparison results with *e* ≤ 1 × 10^−3^; (3) Metagenemark (v3.38) was used to predict the genes, and then HMMSearch (v3.2.1) software was used to compare the protein sequences with the HMM database (VPFS and VFAM), and the comparison results were screened with *e* ≤ 1 × 10^−5^.

### Elimination of False Positives

The candidate virus sequences obtained above were compared with the NT database BLAST (v2.9.0+) and screened at e ≤ 1 × 10^−10^. The sequences not aligned in the previous step were compared with the NR database Diamond (v0.9.10) and screened with *e* ≤ 1 × 10^−3^. NCBI taxonomy data was used to annotate the above-mentioned alignment results. If more than 20% of the alignment results in the first 50 alignment results were non-viral sequences (annotated results were Eukaryota, Bacteria, and Archaea), the sequences were considered to be non-viral sequences, and the rest were considered to be viral sequences. Virus contigs were annotated according to the best hit comparison results of virus contigs and virus-NT (*e* ≤ 1 × 10^−5^).

### Virus Abundance Statistics

Reads were compared with identified virus contigs, and the reads per kilo bases per million reads (RPKM) values of each contig were calculated for comparative analysis between samples.


$$\begin{array}{l} \text{RPKM}=\frac{\text{Contig reads}}{\text{Total mapped reads}\left(\text{millions}\right)\;*\text{ Contig length}\left(\text{KB}\right)} \end{array}$$

Note: (1) Contig reads: number of reads in a contig; (2) Total mapped reads: number of reads in millions; (3) Contig length: contig length, in kbp.

### Gene Prediction

Metagenemark (22) was used to predict the gene sequences of virus contigs, and the number and length of the predicted genes were evaluated.

### Functional Analysis

The predicted gene protein sequence and UniProtKB/Swiss-Prot database sequence of the virus [ViralZone (26), reviewed proteins, https://viralzone.expasy.org/] were used for functional annotation information.

### ASFV Statistical Analysis

The contig on ASFV was identified and compared according to the final virus sequence for analysis. MAFFT (27) software was used to perform multiple comparisons between the ASFV genome and the ASFV reference strain genome.

## Results

### DNA Extraction

**Figure 1:**
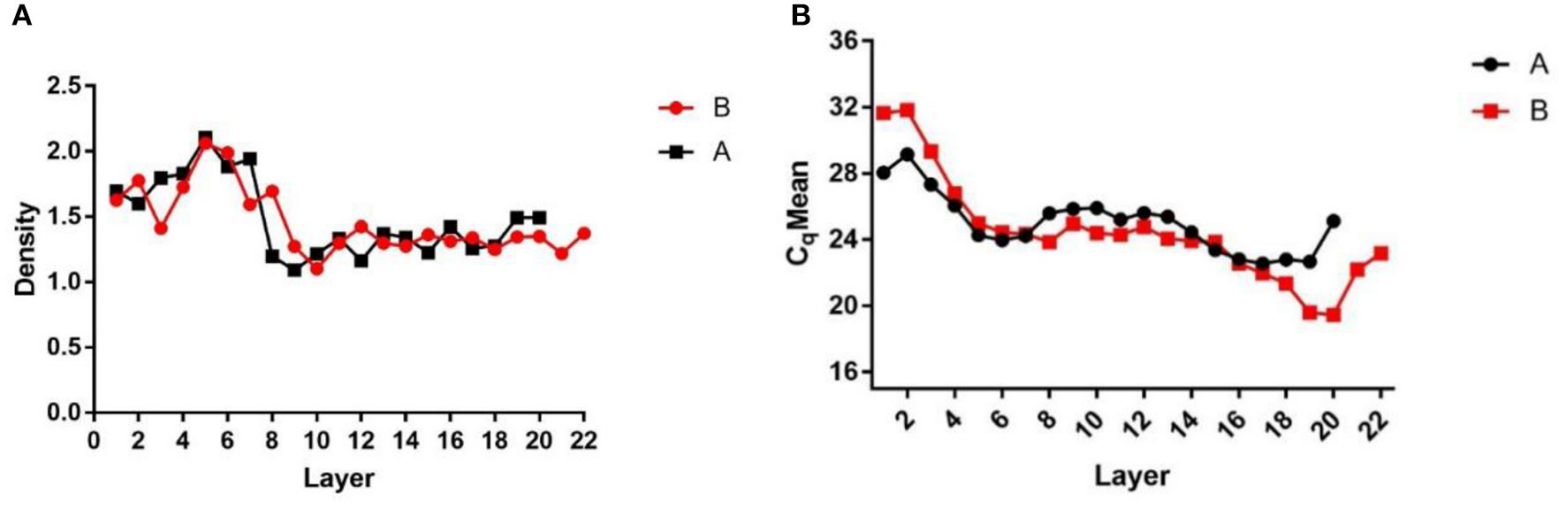
Density and Cq value stratified graphs. **(A)** Density diagram of each liquid layer after overspeed centrifugation; **(B)** Cq value of each liquid layer detected by qPCR after ultracentrifugation. **(A,B)** Are the same two samples of ultracentrifugation.

After centrifugation, the products were layered into Eppendorf tubes, which were weighed before use and after adding each liquid layer. The weights of the liquid layers were recorded and the density of each liquid layer was calculated (Figure 1A) to assess the stratification. The DNA of each liquid layer was extracted with the OMEGA nucleic acid extraction kit, and then qPCR was performed on each liquid layer with specific African swine fever virus primers to determine the virus Cq value in each liquid layer (Figure 1B). The results showed that the Cq value of layer 16–19 was the lowest, and the virions were enriched in layer 16–19. Then, the two liquid layers adjacent to layer 16–19 were mixed, 1 × SM buffer was added, and the virions were fully mixed.

### Quality Control of Sequencing Data

**Figure 2:**
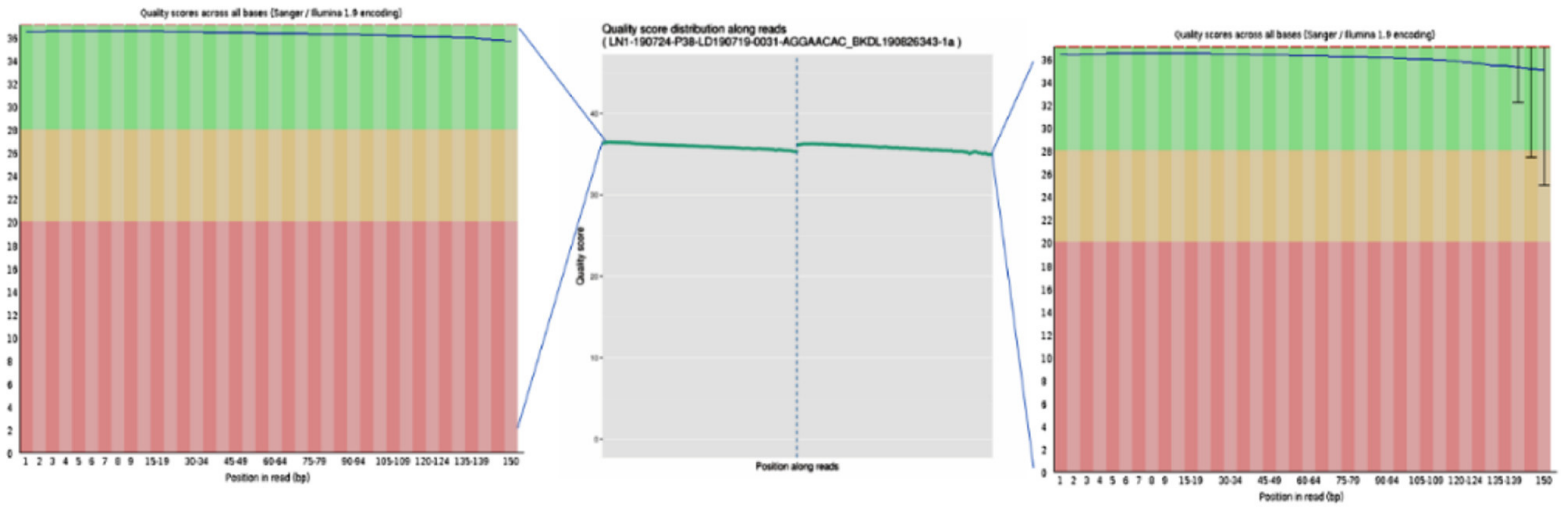
Base quality values of sequencing data from the Illumina sequencing platforms. Quality values are represented by Qphred, where Qphred = −10 × log_10_(e), with e representing the base sequencing error rate. The corresponding relationship between base recognition error rate and Phred score in Illumina Base Calling software is as follows: when Phred score is 10, the base correct recognition rate is 90%; when the Q-score is Q10 and Phred score is 20, the correct base identification rate is 99%; when the Q-score is Q20 and Phred score is 30, the correct base recognition rate is 99.9%; when the Q-score is Q30 and Phred score is 40, the correct base recognition rate is 99.99%; and when the Q-score is Q40, the correct base identification rate is 99.99%. The abscissa represents the position of the base, the ordinate represents the quality of the base at each position, the left graph represents the data before quality control, and the right graph represents after quality control.

To ensure the accuracy of subsequent analysis, SOAPnuke (v2.0.5) software was first used to process the raw data from the machine, and high-quality clean reads were obtained. The quality distribution is shown in Figure 2. The results showed that the average base mass (green line) at all positions was above 30, and the quality of the data was high enough to be used in the following analysis.

### Removal of Host Contamination

To avoid the influence of the host sequence on subsequent analysis, BWA (v0.7.17, default parameter: mem-k30) software was used to compare clean reads with the host database. Sus scrofa was used as the host reference information (accession: NC_010443.5). The comparison results where the length of the comparison was >80% of the total read length were filtered, and then the corresponding sequence was removed. The results showed that 16.5% of clean reads (PE: paired-end) were obtained after host removal, as shown in Table 1.

**Table 1:** Removal of host sequence and virus sequence statistics.

**Sample**	**Raw_reads (PE)**	**Clean reads (PE)**	**Rm host cleam (PE)**	**Rm host cleam (PE) percent (%)**	**Virus reads (PE)**	**Virus percent (%)**
ASFV	50,733,357	34,783,974	5,740,160	16.5	1,109,508	3.19

*(1) Raw reads, the number of original reads, which is the number of double-ended (PE) sequences. (2) Clean reads, the number of filtered reads and the number of double-ended (PE) sequences were counted; (3) Rm host cleam, the number of reads after removing the host, counting the number of double-ended (PE) sequences; (4) The ratio is the result of the comparison with clean reads. (5) Virus reads, the number of reads from the virus was compared, and the number of double-ended (PE) sequences was counted; (6) Virus percent, the ratio of virus sequences to clean reads*.

### Virus Composition Analysis

**Figure 3:**
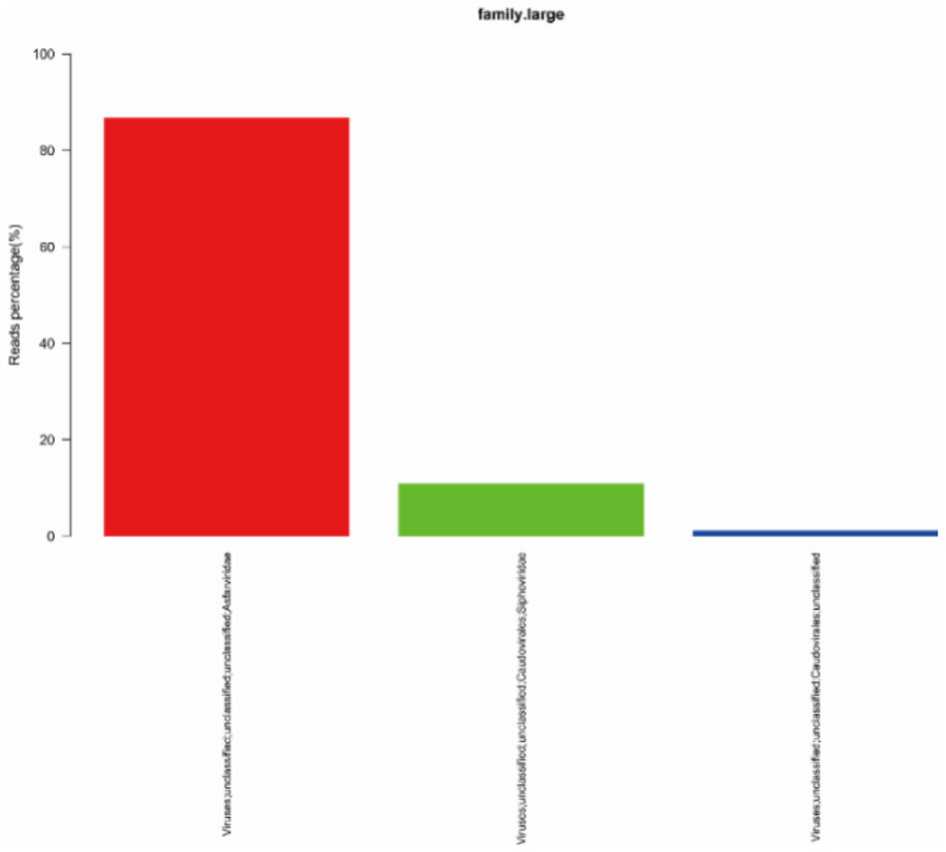
Statistics of the results of horizontal annotation of the viral family. Note: (1) Species with proportion of reads >1% were selected for display; proportion of virus = number of single virus reads/number of total virus reads × 100.

To quickly obtain virus composition information in samples, BWA (v0.7.17, default parameter: mem-k30) software was used to compare clean reads to virus reference data (isolated from NT data); the comparison results were filtered if the length of the comparison was >80% of the total read length. Statistical analysis showed that virus reads (PE) accounted for 3.19% of clean reads (PE), as shown in Table 1. According to the annotation information of the NCBI Taxonomy Database, the virus classification information was counted. To improve the accuracy of the results, comparison results with >5 reads covered were filtered during species annotation. Reads (PE) of the African swine fever virus family (*Asfarviridae*, red column in the figure) accounted for 87.12% of the total reads (PE). *Asfarviridae* had only one ASFV member, so it was not necessary to make annotations at the genus level for the display. The statistical results are shown in Figure 3.

### Data Assembly

**Figure 4:**
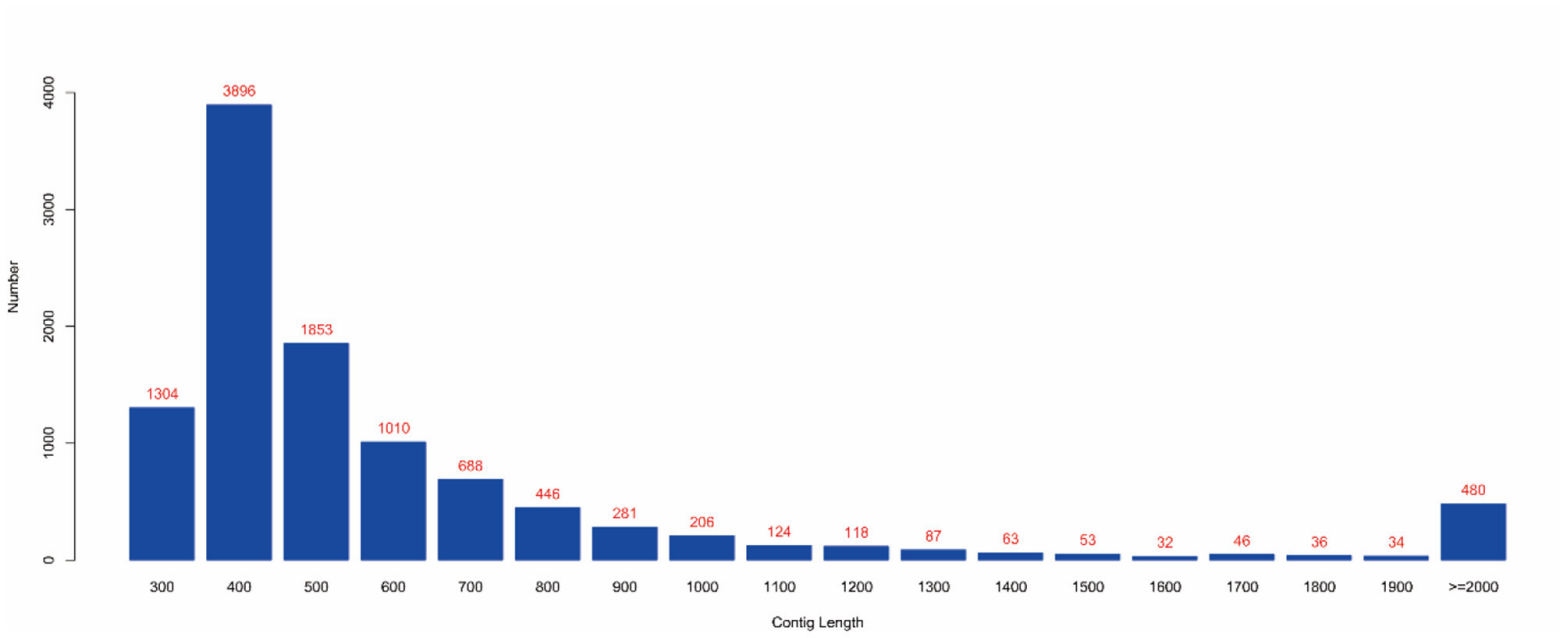
Contig length distribution. (1) The abscissa represents the length of contigs, and the ordinate represents the number of contigs; (2) The red number represents the number of contigs in the length segment.

MEGAHIT (v1.1.2, default parameters: —presets meta-large —min-contig-len 300) software was used to assemble clean data, and BWA (v0.7.17) software was used to compare clean reads with assembly results. The calculated utilization rate of the reads was 75.30%. At the same time, BLAST (v2.9.0+) software was used to align the assembled contigs with the host sequence and to remove the host sequence. The statistical results showed that the maximum number of 400 bp contigs was 3,896 (Figure 4), and the proportion of host sequences was 0.57% (Table 2).

**Table 2:** Removal of host sequence statistics.

**Sample**	**Total_contig**	**Reads_used (%)**	**Host_contig**	**Percent (%)**
A	10,819	75.3	62	0.57

*(1) Sample, name of the sample; (2) READS_USED, reads assembly utilization rate; (3) Host_contig, host sequence; (4) Percentage, proportion of host sequence*.

### Virus Sequence Identification

#### Identification Based on Reference Sequence

**Figure 5:**
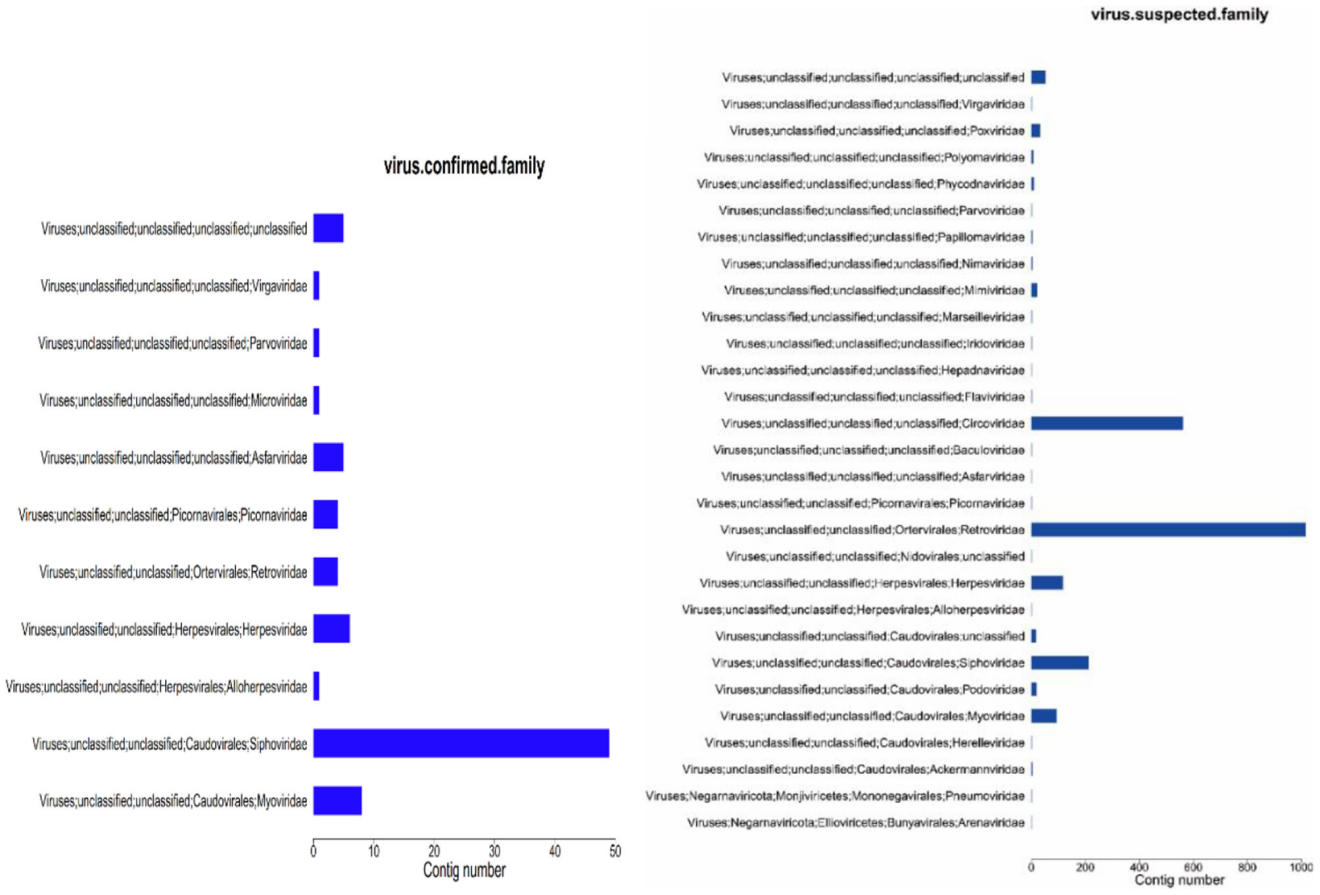
Virus annotation statistics (family level). (1) abscissa: number of contigs; ordinate: comment information.

The obtained contigs were compared to the virus database (isolated from the NT database) using BLAST (v2.9.0+) software. Comparison and screening criteria were as follows. (1) Confirmative: comparison of similarity ≥80%, compare length ≥500 bp, *e* ≤ 1 × 10^−5^, and high reliability; (2) Suspected: did not meet the conditions in (1), compare length ≥100 bp, *e* ≤ 1 × 10^−5^, and low credibility, requiring in-depth analysis and verification; (3) According to NCBI Taxonomy annotation information, the number of phages and other viruses, as well as the types of RNA and DNA, were counted (Tables 3–5, and Figure 5).

**Table 3:** Virus sequence statistics.

**Type**	**Total_base (Mb)**	**Total_num**	**Max_len**	**Min_len**	**N50**	**GC (%)**
Virus.confirmed	0.54	85	114,707	500	40,543	35.69%
Virus.suspected	3.11	2,206	35,033	302	2,882	45.64

*(1) Type, confirmed or suspected contigs; (2) total _base, total number of contig bases; (3) Total_num, number of contigs; Max_len, maximum contig length; Min_len, minimum contig length; (6) N50, N50 value; (7) GC, average content of contig GC*.

**Table 4:** Virus category statistics.

**Type**	**Total**	**Phages (%)**	**Other_virus (%)**
Virus.confirmed	85	63 (74.12%)	22 (25.88%)
virus.suspected	2,206	401 (18.18%)	1805 (81.82%)

*(1) The percentages in brackets are calculated only within confirmed and suspected. Proportion = Virus Class/Total Viruses × 100*.

**Table 5:** Virus category statistics.

**Type**	**Total**	**DNA (%)**	**RNA (%)**
virus.confirmed	85	76 (89.41%)	9 (10.59%)
virus.suspected	2,206	1,178 (53.40%)	1,028 (46.60%)

*(1) The percentages in brackets are calculated only within conception and suspected. Proportion = Virus Class/Total Viruses × 100*.

#### Identification of *De novo* Virus Based on Sequence

Based on the comparison of reference data, there were two defects: (1) Only known viruses could be identified; (2) There were false positives in the results. To reduce false positives and identify unknown viruses, new methods were used to further identify virus sequences by combining multiple databases (Table 6). The analysis process is described in Section 2.7.

**Table 6:** *De novo* virus identification result statistics.

**Sample**	**Total_base (Mb)**	**Total_** **num**	**Max_len**	**Mix_len**	**N50**	**GC**
Novel.viral.contig.final	0.44	327	114,707	302	2,626	51.80%

*(1) Total_base, total number of contigs; (2) Total_num, number of contigs; Max_len, maximum contig length; Min_len, minimum contig length; (5) N50, N50 value; (6) GC, average content of contig GC*.

#### Comparison of the Two Methods

**Figure 6:**
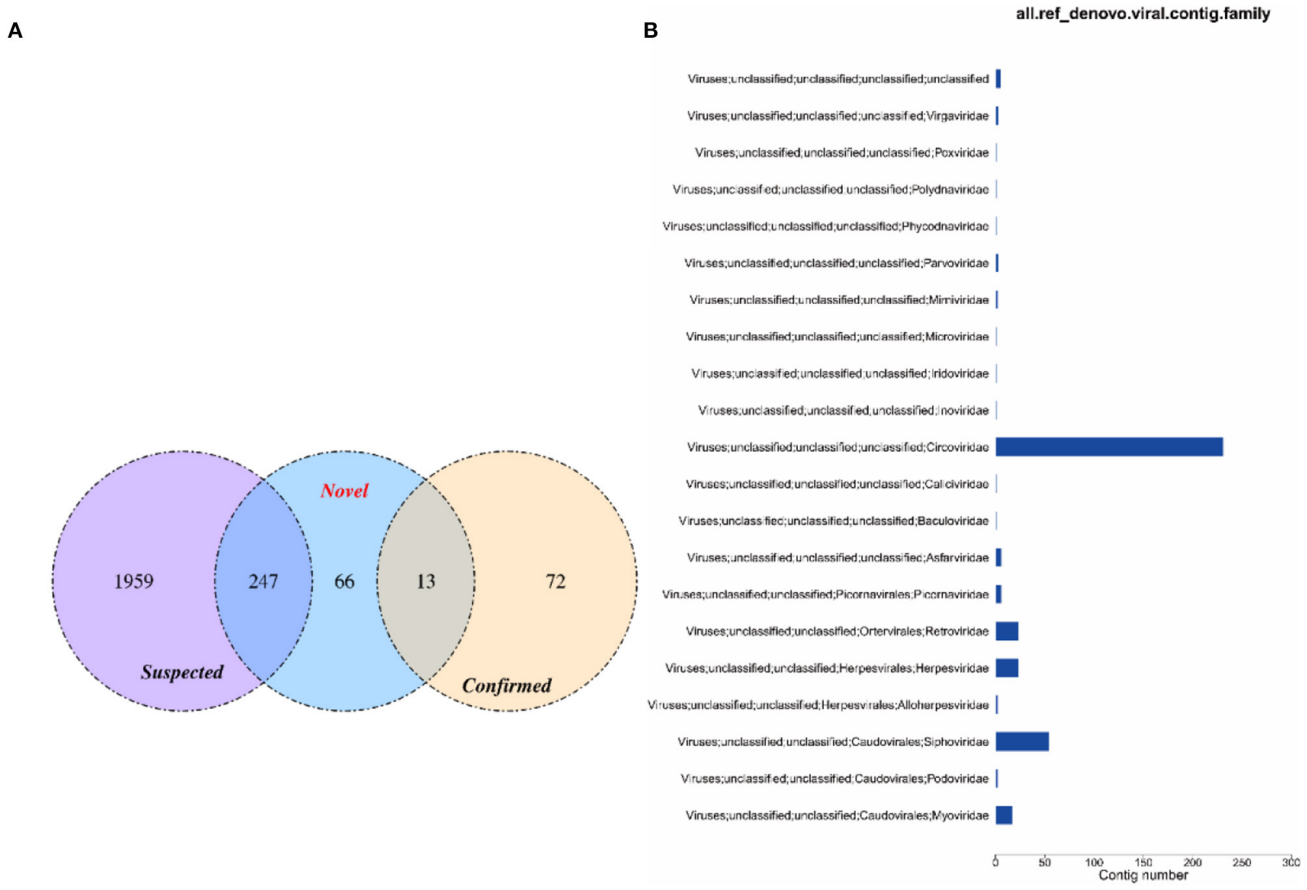
Venn and annotated statistical diagrams of the virus sequence. **(A)** Comparison of the number of contigs obtained by different methods. (1) Novel method to obtain virus sequence; (2) Confirmed: confirmed virus sequence; (3) Suspected sequence of a virus. **(B)** Statistics of final viral sequence annotations (family level).

The virus sequences obtained by the two methods were compared, and Venn diagram were drawn. Then, the confirmed and novel virus sequences (Section 3.6.1 and 3.6.2) were combined as the final virus sequences (Figure 6 and Table 7).

**Table 7:** Virus contig statistics.

**Sample**	**Total_base (Mb)**	**Total_** **num**	**Max_** **len**	**Mix_len**	**N50**	**GC**
All.ref_denovo.viral.contig	0.77	398	114,707	302	9,767	43.88%

*(1) Total_base, total number of contigs; (2) Total_num, number of contigs; Max_len, maximum contig length; Min_len, minimum contig length; (5) N50, N50 value; (6) GC, average content of contig GC*.

### Virus Abundance Analysis

BWA (v0.7.17, default parameter: mem-k 30) software was used to compare the host-removed clean reads with the obtained virus contigs, and the proportion of virus reads was calculated when the filter comparison length was lower than 80% of the total read length. The RPKM value of each viral contig was calculated. The maximum RPKM value was 85,317.57 and the minimum RPKM value was 224.62 (Table 8).

**Table 8:** Comparison results of clean reads and virus contigs.

**Sample**	**Clean_reads (PE)**	**Mapped_reads (PE)**	**Percent (%)**
A	34,783,974	1,351,282	3.88

*(1) Sample, name of the sample; (2) Clean reads, the number of filtered reads, which counted the number of double-ended (PE) sequences; (3) Mapped_reads, the number of double-ended (PE) sequences was calculated by comparing the number of contig reads; (4) Percentage, ratio of mapped reads to clean reads*.

### Gene Prediction

Metagenemark (v3.38) software was used to predict the gene corresponding to virus contigs, and sequences with gene nucleic acid length >150 bp were filtered. The statistical results are presented in Table 9.

**Table 9:** Gene prediction result statistics.

**Sample**	**Total_** **base (Mb)**	**Total_** **num**	**Max_len**	**Min_len**	**N50**	**GC**
viral.contig.gene_nucl	0.45	552	7,431	153	1,068	36.79%

*(1) TOTAL_BASE, total number of bases in a sequence; (2) Total_num, number of entries in a sequence; (3) Max_len, the maximum length; (4) Min_len, the minimum length; (5) N50, N50 value; (6) GC, average GC content*.

### Functional Analysis

**Figure 7:**
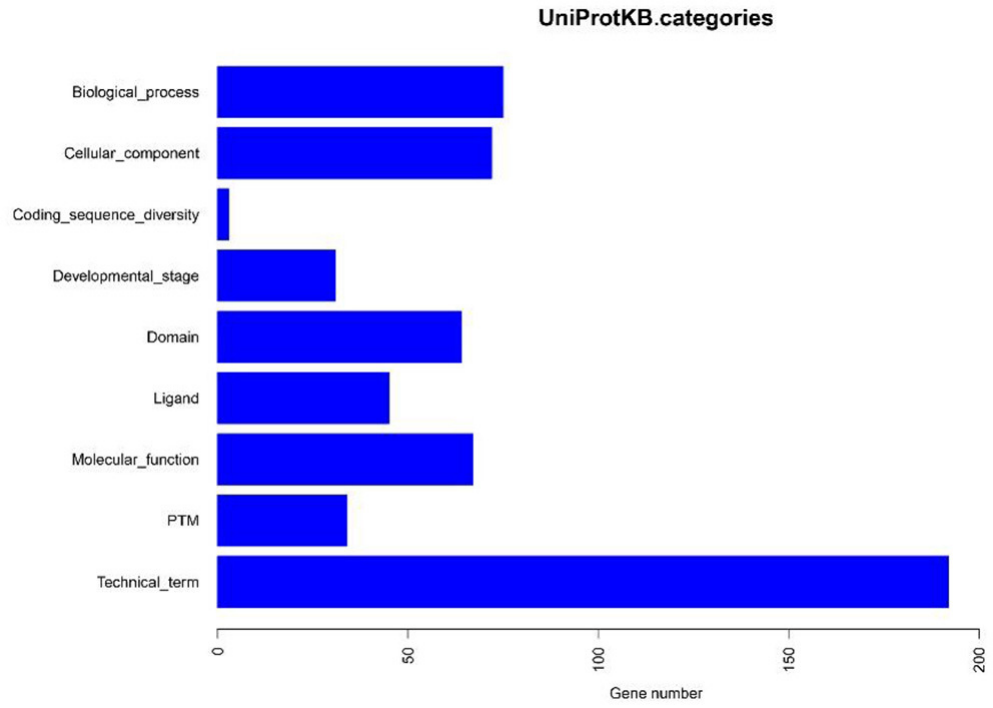
UniProtKB category statistics.

Based on the BLASTP (v2.9.0+) software, the gene sequences were compared with those collected from the UniProtKB/SWISS-PROT database (https://viralzone.expasy.org/). The best hit screened with *e* < 1 × 10^−3^ was compared to obtain the function information of the virus. The results showed that the percentage of genes in the database accounted for 37.14% of the total gene number (Table 10). Functional genes annotated by UniProtKB category statistics included biological processes, cell components, and molecular functions (Figure 7). The polymerase gene number statistics are shown in Table 11.

**Table 10:** Gene annotation rate statistics.

**Total.gene.num**	**gene.vs.UniProtKB.num**	**Percent (%)**
552	205	37.14

*(1) Total gene num, total number of genes predicted; Num, number of genes in UniProtKB/SWISS-PROT ViralZone database; (3) Percentage, percentage of the total number of genes compared to the database*.

**Table 11:** Polymerase gene number statistics.

**Type**	**Number**
DNA-directed_DNA_polymerase	4
DNA-directed_RNA_polymerase	6
RNA-directed_DNA_polymerase	0
RNA-directed_RNA_polymerase	2
Other. polymerase	5

*Other. polymerase includes the following categories: Polymeres, polymeres that can be cleaved by polymerases, helicases, etc.; Polymeres that can be cleaved by polymerases, helicases, etc.; Co-factor of polymerase; Polymerase-related proteins*.

### ASFV Analysis

**Figure 8:**
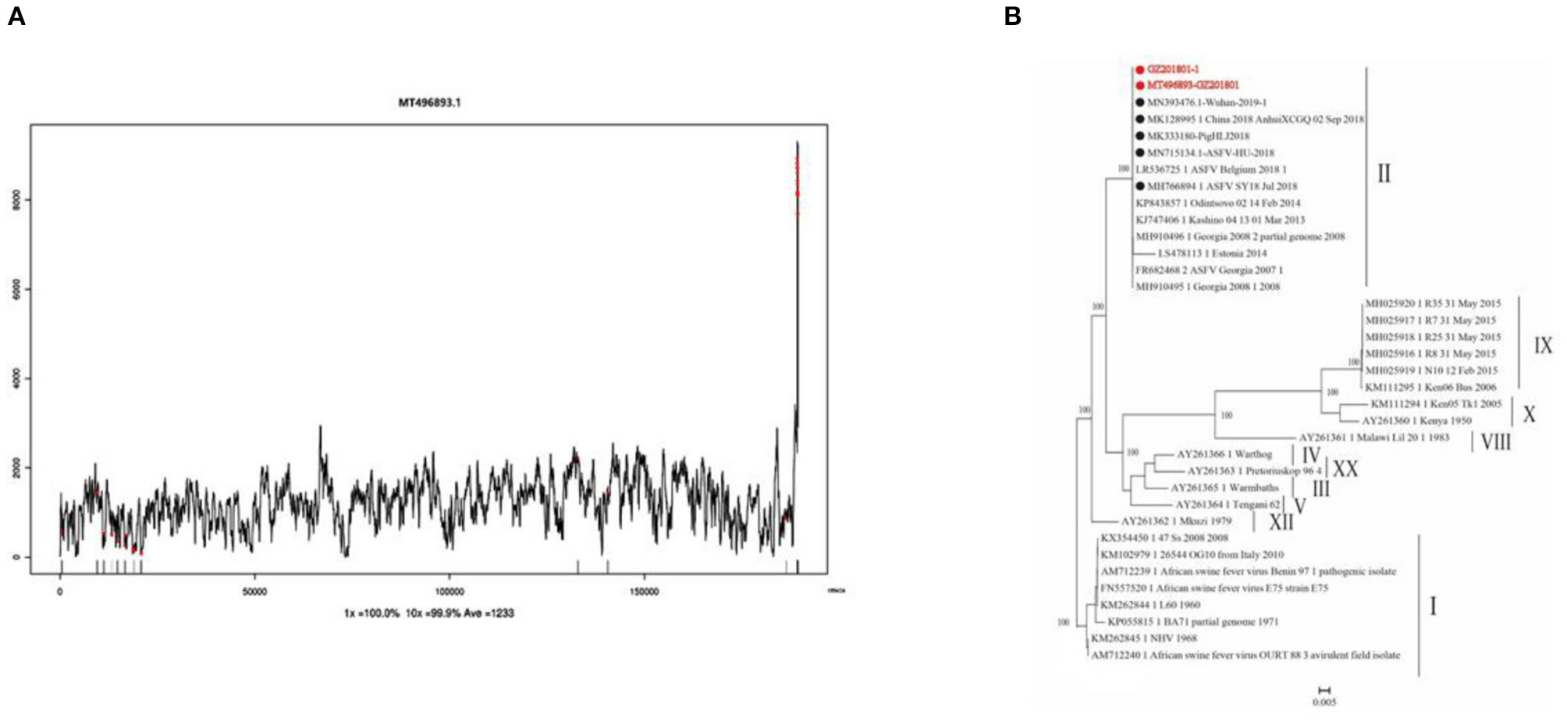
Sequencing depth map and genome-wide phylogenetic tree. **(A)** Sequencing depth map of African swine fever virus. **(B)** Genome-wide phylogenetic tree of the GZ201801 strain.

Clean reads were compared to the reference sequence MT496893.1 by using BWA and SamTools software, and a consensus sequence was obtained. The average sequencing depth was 1,233. According to the final virus sequence in Section 3.6.3, the ASFV contig was found and compared with the reference sequence MT496893.1. Sequences were filtered using similarities <90% and lengths <500 bp; the results showed that there were five matched contigs, and the longest was 114,708 bp. After overlapping, correcting, and assembling with the MT496893.1 sequence, the whole genome sequence, named GZ201801-1, was obtained with a length of 188,035 bp. However, the assembled genome GZ201801-1 was 1,358 bp shorter than GZ201801. To construct the phylogenetic tree, representative ASFV sequences from GenBank were selected, including genotype II ASFV strain (*n* = 12), genotype I ASFV strain (*n* = 8), genotype II ASFV strain (*n* = 6), genotype I ASFV strain (*n* = 2), and genotype VIII, IV, XX, III, V, and XII ASFV strains (each one). Phylogenetic analysis of the entire viral genome was performed using the NJ method in Mega X, and the phylogenetic tree bootstrap value was set to 1,000 (Table 12 and Figure 8).

**Table 12:** Statistical analysis of total length and similarity of ASFV.

**Contig**	**Identity (%)**	**Alignment_length**
ASFV|contig_2,509	99.888	896
ASFV|contig_2,689	99.541	1,089
ASFV|contig_3,673	100	513
ASFV|contig_5,899	99.97	72,239
ASFV|contig_9913	99.997	114708

*(1) Contig, ID number; (2) Identity (%), similarity ratio; (3) ALIGNMENT_LENGTH, the length of alignment with the reference sequence*.

## Discussion

The ASFV genome is relatively conserved and its natural variation is very slow, but interaction with the host and stimulation by environmental factors can accelerate the transmission of ASFV (28). Given the current lack of genome-wide information, it is necessary to promote the whole-genome sequencing of ASFV. In recent years, the development of sequencing technology has provided technical support for whole genome sequencing, including the macro virus group method (29).

The macro virus group (virome) method directly uses the genetic material of all viruses in the samples for analysis. First, virus particles are enriched, the genome sequence information is obtained, and then the composition and relative abundance of all viruses are identified. This method is a powerful means for the discovery of new viruses and the early detection and control of virus infection. It is of great use in the study of the origin and evolutionary patterns of viruses, genetic diversity, geographical distribution, and the relationship between viruses and their hosts. In the early stage, we used first-generation sequencing to sequence the whole genome of the Guangdong outbreak ASFV, and submitted the sequence to GenBank (serial number: MT496893.1). Due to the time-consuming characteristic of first-generation sequencing, we improved the sequencing analysis method based on the macro virus group (virome) method. Previous studies used a density gradient buffer of Percoll, sucrose, and cesium chloride (30), whereas our steps of using Optiprep separation and improved centrifugation effectively increased the proportion of virus samples provided more effective data for subsequent analysis. Optiprep Separation liquid is characterized by high density, low viscosity, and low permeability. Continuous or discontinuous isotonic gradient solutions can be formed when appropriate concentrations of buffers or basic media are added to separate various cells, nuclei, organelles, and lipoproteins. Compared with the traditional separation solutions (such as sucrose, Percoll, and cesium chloride), it not only has good separation and purification effect, but also has no effect on the life activities of various cells and organelles. In this experiment, the virions in solution were purified and concentrated by overspeed centrifugation of spleens, and the enrichment layer of the virions was then determined by qPCR to obtain higher purity virions for viral DNA extraction. High-purity samples yielded high-quality data for downstream assembly and analysis. Data quality control, host sequence removal, virus classification, data assembly, virus sequence identification, statistical analysis of virus abundance, gene prediction, and functional analysis were performed. Due to the high error rate of second-generation sequencing methods, we used different virus identification methods to restore the diversity and abundance of viruses in the sample as much as possible.

The results of data quality control showed that the average base mass at all positions was above 30, indicating high data quality. A total of 1,351,282 reads (3.88% of the 34,783,974 reads) were mapped to the entire genome region of the MT496893.1 strain, with an average depth of 1,233. Finally, the MT496893.1 strain was used as a reference for genome correction and assembly, and a 188,035 bp genome was finally obtained. If tissue can be ground and supercentrifuged, the high purity and concentration of virus particles can be theoretically stratified for second-generation sequencing, and virus isolation is not required, which will shorten the time to obtain the results (31). Using clinical samples and overspeed centrifugation to obtain high purity of virus particles, we confirmed that we could get high quality sequencing data; this was advantageous to the subsequent assembly and analysis, due to more timely whole genome sequencing of the outbreak strain, facilitating the analysis of the origin and evolution of ASFV, genetic diversity, and geographical distribution. In summary, our results confirm the utility of virome sequencing as a non-culture direct sequencing method for ASFV genomes from PCR-positive clinical tissues.

## Data Availability Statement

The original contributions presented in the study are included in the article/supplementary material, further inquiries can be directed to the corresponding authors.

## Author Contributions

HW and GZ: conceived and designed the experiments. CJ, JJ, and YW: performed the experiments. YCh, YW, ZM, MC, HY, GLi, and LG: sample collection. CJ, CQ, YCh, and GLi: analyzed the data. CJ and HW: contributed to the writing. All authors have read and approved the final manuscript.

## Funding

The Key-Area Research and Development Program of Guangdong Province (2019B020211003 and 2019B020211005). National Natural Science Foundation of China (31941004) and China Agriculture Research System of MOF and MARA.

## Conflict of Interest

CJ, YW, YCh, GZ, and HW were employed by company Shenzhen Kingkey Smart Agriculture Times Co., Ltd. The remaining authors declare that the research was conducted in the absence of any commercial or financial relationships that could be construed as a potential conflict of interest.

## Publisher's Note

All claims expressed in this article are solely those of the authors and do not necessarily represent those of their affiliated organizations, or those of the publisher, the editors and the reviewers. Any product that may be evaluated in this article, or claim that may be made by its manufacturer, is not guaranteed or endorsed by the publisher.
